# Incidence and risk factors of proximal junctional kyphosis in adolescent idiopathic scoliosis after correction surgery: a meta-analysis and systematic review

**DOI:** 10.1186/s13018-024-04638-7

**Published:** 2024-04-02

**Authors:** XingHua Ji, LinDong Wei, ZeJun Xing, YuChen Duan

**Affiliations:** 1 Shanxi Bethune Hospital, Shanxi Academy of Medical Sciences. Tongji Shanxi Hospital, Third Hospital of Shanxi Medical University, Taiyuan, 030032 China; 2 Tongji Hospital, Tongji Medical College Huazhong University of Science and Technology, Wuhan, 430030 China

**Keywords:** Adolescent idiopathic scoliosis, Proximal junctional kyphosis, Risk factor, Meta-analysis

## Abstract

**Aim:**

To analyze the risk factors of proximal junctional kyphosis (PJK) after correction surgery in patients with adolescent idiopathic scoliosis (AIS).

**Methods:**

PubMed, Medline, Embase, Cochrane Library, Web of Science, CNKI, and EMCC databases were searched for retrospective studies utilizing all AIS patients with PJK after corrective surgery to collect preoperative, postoperative, and follow-up imaging parameters, including thoracic kyphosis (TK), lumbar lordosis (LL), proximal junctional angle (PJA), the sagittal vertical axis (SVA), pelvic incidence (PI), pelvic tilt (PT), pelvic incidence–lumbar lordosis (PI–LL), sacral slope (SS), rod contour angle (RCA) and upper instrumented vertebra (UIV).

**Results:**

Nineteen retrospective studies were included in this meta-analysis, including 550 patients in the intervention group and 3456 patients in the control group. Overall, sex (OR 1.40, 95% CI (1.08, 1.83), *P* = 0.01), larger preoperative TK (WMD 6.82, 95% CI (5.48, 8.16), *P* < 0.00001), larger follow-up TK (WMD 8.96, 95% CI (5.62, 12.30), *P* < 0.00001), larger postoperative LL (WMD 2.31, 95% CI (0.91, 3.71), *P* = 0.001), larger follow-up LL (WMD 2.51, 95% CI (1.19, 3.84), *P* = 0.0002), great change in LL (WMD − 2.72, 95% CI (− 4.69, − 0.76), *P* = 0.006), larger postoperative PJA (WMD 4.94, 95% CI (3.62, 6.26), *P* < 0.00001), larger follow-up PJA (WMD 13.39, 95% CI (11.09, 15.69), *P* < 0.00001), larger postoperative PI–LL (WMD − 9.57, 95% CI (− 17.42, − 1.71), *P* = 0.02), larger follow-up PI–LL (WMD − 12.62, 95% CI (− 17.62, − 7.62), *P* < 0.00001), larger preoperative SVA (WMD 0.73, 95% CI (0.26, 1.19), *P* = 0.002), larger preoperative SS (WMD − 3.43, 95% CI (− 4.71, − 2.14), *P* < 0.00001), RCA (WMD 1.66, 95% CI (0.48, 2.84), *P* = 0.006) were identified as risk factors for PJK in patients with AIS. For patients with Lenke 5 AIS, larger preoperative TK (WMD 7.85, 95% CI (5.69, 10.00), *P* < 0.00001), larger postoperative TK (WMD 9.66, 95% CI (1.06, 18.26), *P* = 0.03, larger follow-up TK (WMD 11.92, 95% CI (6.99, 16.86), *P* < 0.00001, larger preoperative PJA (WMD 0.72, 95% CI (0.03, 1.41), *P* = 0.04, larger postoperative PJA (WMD 5.54, 95% CI (3.57, 7.52), *P* < 0.00001), larger follow-up PJA (WMD 12.42, 95% CI 9.24, 15.60), *P* < 0.00001, larger follow-up SVA (WMD 0.07, 95% CI (− 0.46, 0.60), *P* = 0.04), larger preoperative PT (WMD − 3.04, 95% CI (− 5.27, − 0.81), *P* = 0.008, larger follow-up PT (WMD − 3.69, 95% CI (− 6.66, − 0.72), *P* = 0.02) were identified as risk factors for PJK.

**Conclusion:**

Following corrective surgery, 19% of AIS patients experienced PJK, with Lenke 5 contributing to 25%. Prior and post-op measurements play significant roles in predicting PJK occurrence; thus, meticulous, personalized preoperative planning is crucial. This includes considering individualized treatments based on the Lenke classification as our future evaluation standard.

**Supplementary Information:**

The online version contains supplementary material available at 10.1186/s13018-024-04638-7.

## Introduction

The most prevalent type of scoliosis is adolescent idiopathic scoliosis (AIS), which affects more girls than boys globally and has a prevalence of 0.47–5.2% [1]. Tortuosity occurs during pubertal development, and it is manifested as transverse and horizontal torsion deformity of the thoracic and/or lumbar vertebrae. The severity of the deformity is inversely proportional to the overall balance control ability of the spine [2]. Severe AIS may lead to razor back deformity, intervertebral disc degeneration, cervical kyphosis, and late decompensation [3]. Moreover, it can even lead to cardiopulmonary insufficiency and irreversible nerve damage [4], as well as affect [5]. The current treatment methods include surgery and conservative orthosis treatment, whereby the posterior approach is the most common surgical procedure. A long-term follow-up study of AIS has shown [6] that spinal correction surgery can preserve the good balance of the spine while maintaining aesthetics and improving the quality of life of patients.

After spinal correction, there is a chance of early surgical complications. Proximal junctional kyphosis is one of the most typical consequences (PJK) [7], with an incidence ranging from 9.2 to 61.7% [8]. Proximal junctional kyphosis (PJK) was defined as the final proximal junctional sagittal Cobb Angle (PJA) between the lower-end plate of the upper vertebra (UIV) and the upper-end plate of UIV + 2, ≥ 10° compared to the preoperative measurement [9]. The usual manifestation of PJK is a kyphotic change in the disc space above the fusion [10], leading to impaired sagittal balance, vertebral collapse, and neuropathy. In more severe cases, revision surgery is required [11]. The occurrence of junctional kyphosis after orthopedic surgery is closely related to multiple AIS risk factors, including advanced age, osteopenia, obesity, and the severity of preoperative sagittal imbalance and intraoperative correction [12], but it has not been fully elucidated.

In order to prevent PJK, lessen the long-term consequences of spinal deformity surgery, and improve the physical function of patients by identifying the risk factors of complications, this meta-analysis has been carried out on patients with AIS to investigate the incidence and risk factors of PJK after orthopedic surgery.

## Materials and methods

A research protocol was registered through PROSPERO: International Prospective Register of Systematic Reviews (protocol CRD42023416848) and completed conforming to the Preferred Reporting Items for Reviews and Meta-Analyses (PRISMA) guidelines for systematic review.

### Literature search

Studies were identified through a systematic literature search of online databases: PubMed, Medline, Embase, Cochrane Library, Web of Science, CNKI, and EMCC. An electronic database search for full-text articles and published abstracts from the inception of each database to April 2023 was conducted. The search was not limited by factors such as language, geographic origin, date of publication, or study type. For database searches, the following main keywords were the following text words: “Adolescent Idiopathic Scoliosis” OR “AIS” AND “Proximal junctional kyphosis” OR “PJK.”

### Inclusion and exclusion criteria

All available studies were included in patients with AIS and PJK who underwent corrective surgery. PJK was defined by the presence of two criteria: (1) a proximal junction sagittal Cobb angle of ≥ 10° and (2) a postoperative proximal junction sagittal Cobb angle at least 10° greater than the measurement preoperatively [9]. Inclusion criteria: (1) underwent the same posterior approach; (2) divided into PJK groups and non-PJK groups; (3) sufficient data. Exclusion criteria: (1) patients with prior spinal surgery, anterior release, congenital scoliosis, incomplete spine, and those related to syndromes including Ehlers-Danlos Syndrome were excluded; (2) no available data; (3) duplicate report, pure summary, case report, and conference paper.

### Data extraction

Basic demographic data were gathered, including age, sex, body mass index, and follow-up time. A full-spine frontal and lateral radiography study was completed preoperatively, postoperatively, and at the final follow-up. Radiographic parameters included thoracic kyphosis (TK), lumbar lordosis (LL), proximal junctional angle (PJA), the sagittal vertical axis (SVA), pelvic incidence (PI), pelvic tilt (PT), pelvic incidence–lumbar lordosis (PI–LL), sacral slope (SS), rod contour angle (RCA) and upper instrumented vertebra (UIV).

### Study selection and data extractions

From the literature search, 322 abstracts of studies were retrieved and independently screened for inclusion. The information extracted included study general study (title, author and year), study characteristics (Lenke type, country, type of study design and follow-up month), and the number of cases (Table 1). 279 articles were excluded by reading the abstracts for any one of the following reasons: nonrelevant material, articles with unavailable data and duplicate studies. Therefore, 19 full-text articles were reviewed for inclusion. All studies met the inclusion criteria and were subsequently reviewed and analyzed.

**Table 1:** General features included in the study

Study	Lenke type	Country	Research type	Follow-up month (m)	Cases	
					PJK	Non-PJK
Amanullah 2022 [13]	–	USA	Retrospective study	Minimum 24	8	17
Boeckenfoerde 2022 [14]	–	Switzerland	Retrospective study	Minimum 27	30	139
Chen 2019 [15]	5	China	Retrospective study	Minimum 24	12	21
Chen J 2021 [16]	5	China	Retrospective study	Minimum 24	15	20
Clément 2021 [17]	1,2,3,4,6	France	Retrospective study	Minimum 24	102	468
Ferrero 2018 [18]	1,2	France	Retrospective study	Minimum 24	57	308
Ghailane 2017 [19]	1,2,3,4,6	France	Retrospective study	Average 18 (range, 10–26)	5	45
Helgeson 2010 [20]	–	USA	Retrospective study	Minimum 24	8	275
Hu 2022 [21]	5C	China	Retrospective study	Minimum 24	23	75
Kim 2007 [22]	–	USA	Retrospective study	Minimum 24	111	299
Kim 2021 [23]	–	Switzerland	Retrospective study	Minimum 60	7	62
Li 2020 [24]	5	China	Retrospective study	Minimum 12	10	34
Lonner 2017 [25]	–	USA	Retrospective study	Minimum 24	60	791
Ogura 2021 [26]	1,2,3	USA	Retrospective study	Minimum 12	15	330
Pahys 2018 [27]	–	USA	Retrospective study	Minimum 24	6	348
Wang 2020 [28]	5	China	Retrospective study	Minimum 24	12	40
Wang J 2020 [29]	–	China	Retrospective study	Minimum 18	21	75
Zhao 2018 [30]	5	China	Retrospective study	Minimum 24	35	52
Zhou 2021 [31]	5	China	Retrospective study	Minimum 24	13	57

The authors independently implemented the Newcastle–Ottawa Scale Assessment Scale (NOSSA) to assess for the following biases: selection, comparability, and outcome (Table 2). Consequently, the quality of evidence for this study was deemed high.

**Table 2:** Results of bias risk assessment in included case–control studies

Study	Selection				Comparability	Exposure			Scores
	Adequate definition of cases	Representativeness of cases	Selection of controls	Definition of controls	Control for important factor	Ascertainment of exposure	Same methods of ascertainment for cases and controls	Non-response rate	
Amanullah 2022	1	1	1	1	2	1	1	1	9
Boeckenfoerde 2022	1	1	1	1	2	1	1	1	9
Chen 2019	1	1	1	1	2	1	1	1	9
Chen J 2021	1	1	1	1	2	1	1	1	9
Clément 2021	1	1	1	1	2	1	1	1	9
Ferrero 2018	1	1	1	0	2	1	1	1	8
Ghailane 2017	1	0	1	0	2	1	1	1	7
Helgeson 2010	1	1	0	1	2	1	1	1	8
Hu 2022	1	0	1	1	2	1	1	1	8
Kim 2007	1	1	0	1	2	1	1	1	8
Kim 2021	1	1	1	1	2	1	1	1	9
Li 2020	1	1	1	1	2	1	1	1	9
Lonner 2017	1	1	0	1	2	1	1	1	8
Ogura 2021	1	1	1	1	2	1	1	1	8
Pahys 2018	1	1	0	1	2	1	0	1	8
Wang 2020	1	1	1	1	2	1	1	1	9
Wang J 2020	1	1	1	1	2	1	0	1	8
Zhao 2018	1	1	0	1	2	1	1	1	8
Zhou 2021	1	1	1	1	2	1	0	1	8

### Quality assessment and statistical analysis

All meta-analyses were performed using Review Manager 5.3 (Cochrane Collaboration, Oxford, UK). Continuous and dichotomous variables were analyzed using weighted mean differences (WMDs) and risk ratios (ORs) with 95% confidence intervals (CIs), respectively. The statistical heterogeneity was quantified using the I^2^. The random-effects model was used if there was heterogeneity between studies (I^2^ > 50%); otherwise, the fixed-effects model was used (I^2^ < 50%). The random or fixed-effects model is determined by comparing the significant difference in the combination graph (Fig. 6, etc.).

## Results

### Selection of studies for inclusion in the systematic review and meta-analysis

The detailed study selection process is documented in the PRISMA (Preferred Reporting Items for Systematic Reviews and Meta-Analyses) flowchart. The search strategy is illustrated in Fig. 2. The initial systematic literature search yielded 323 publications. The full texts of 43 publications were examined, and 19 investigations were discarded.

**Fig. 1:**
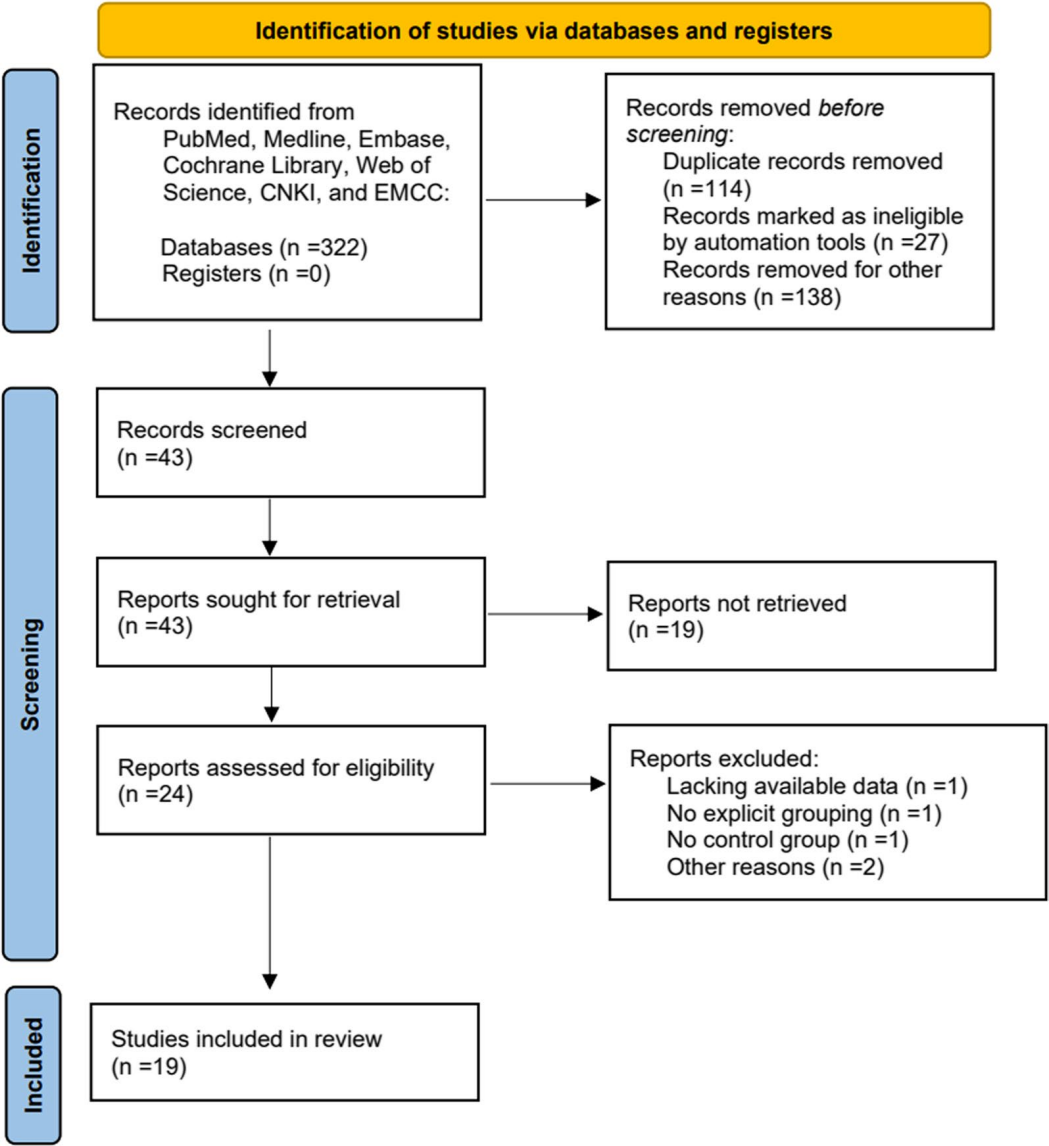
Literature screening flow chart and results

5 papers were ineligible for the following reasons: 1 paper did not provide complete data for this meta-analysis, 1 paper without a control group, 1 paper with no explicit grouping, and 2 papers for other reasons. 19 studies that satisfied the screening requirements were selected for this meta-analysis (Fig. 1).

### Risk factors

**Fig. 2:**
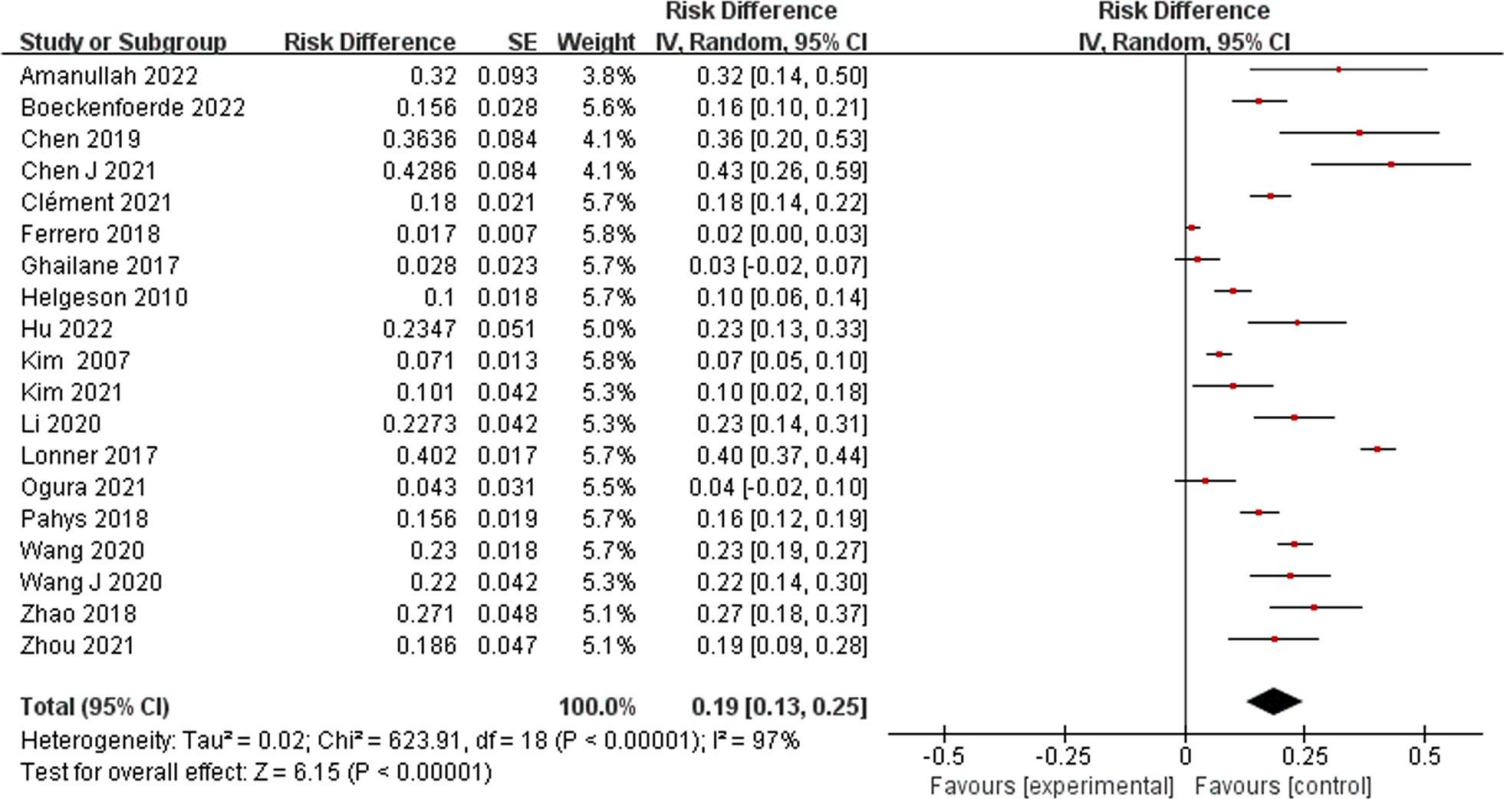
Pooled incidence of proximal junctional kyphosis

**Fig. 3:**
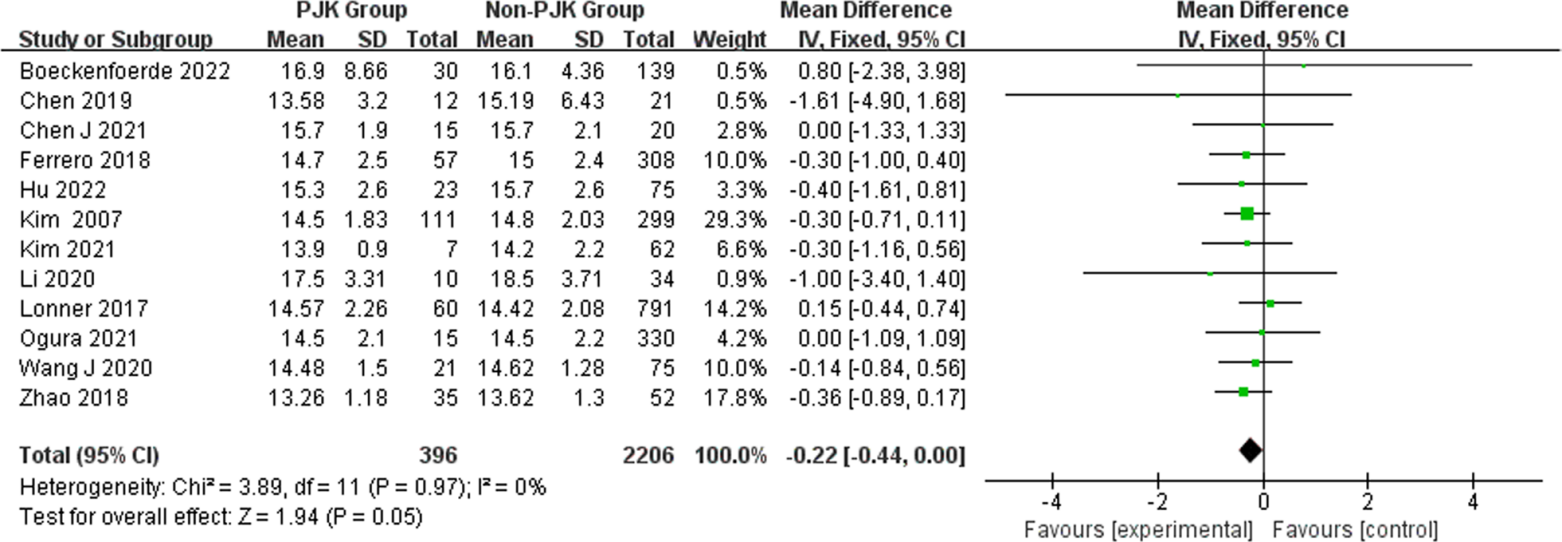
Forest plot of age between the proximal junctional kyphosis (PJK) group and the non-PJK

**Fig. 4:**
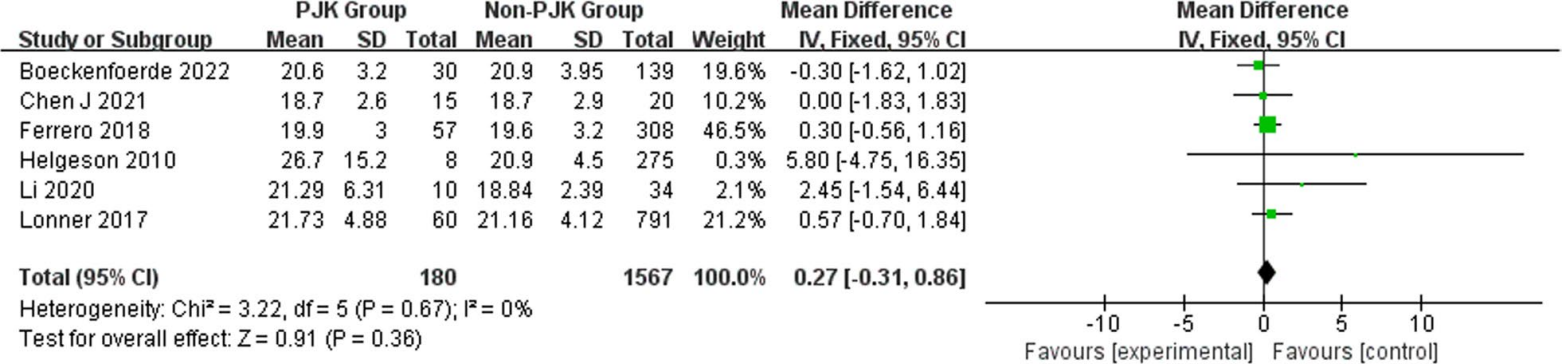
Forest plot of BMI between the proximal junctional kyphosis (PJK) group and the non-PJK

**Fig. 5:**
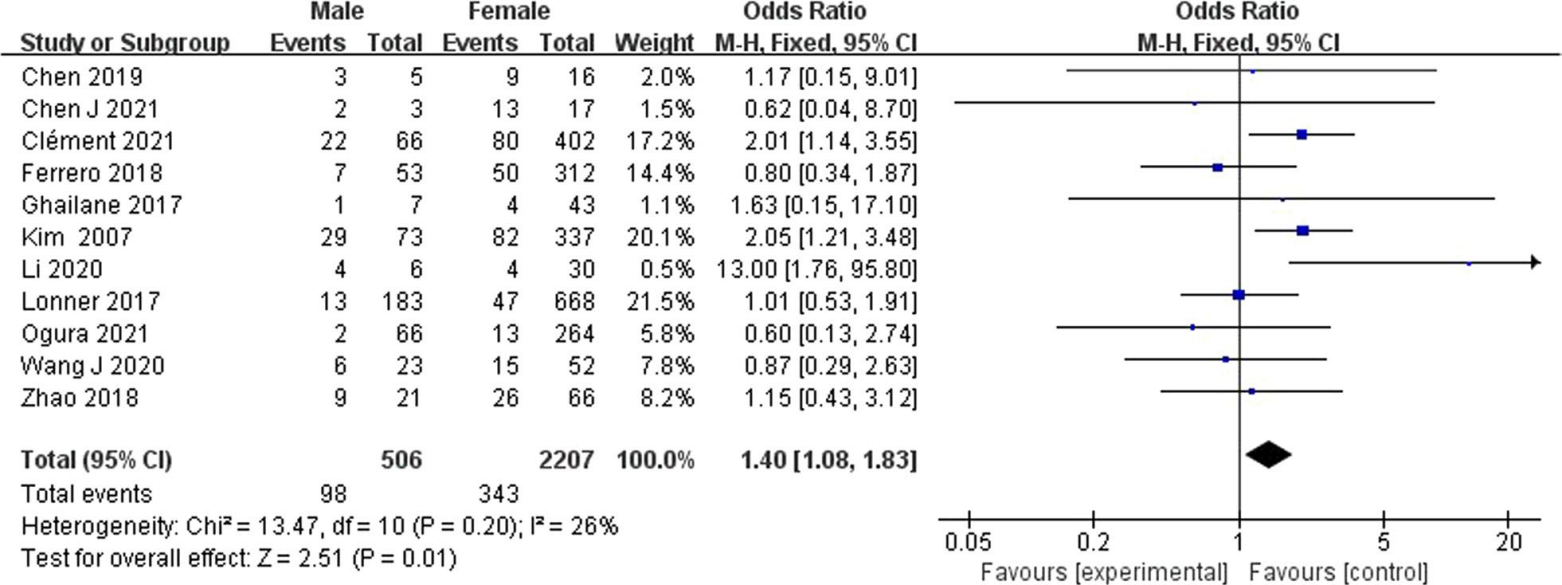
Forest plot of proximal junctional kyphosis between the male and female groups

A total of 550 patients with AIS had PJK after undergoing correction surgery. The overall pooled incidence of PJK was 19% (95% CI 13–25%) based on the 19 studies (Fig. 2). Our results showed that age (WMD − 0.22, 95% CI (− 0.44, 0.00), *P* = 0.05) (Fig. 3) and body mass index (WMD 0.27, 95% CI (− 0.31, 0.86), *P* = 0.36) (Fig. 4) were not significantly associated with PJK. Sex (OR 1.40, 95% CI (1.08, 1.83), *P* = 0.01) (Fig. 5) is significantly associated with PJK.

**Fig. 6:**
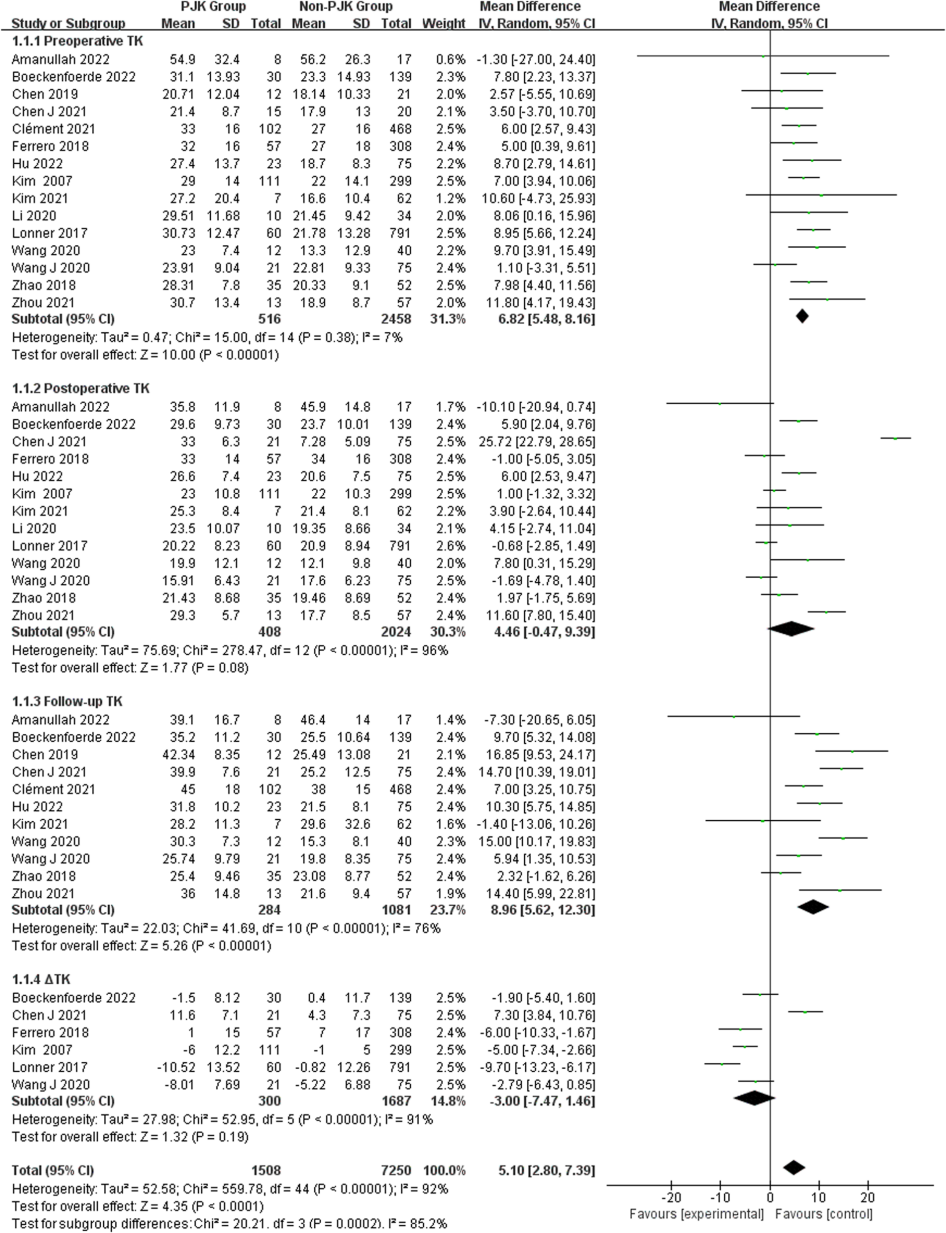
Forest plot of TK between proximal junctional kyphosis (PJK) and non-PJK groups

**Fig. 7:**
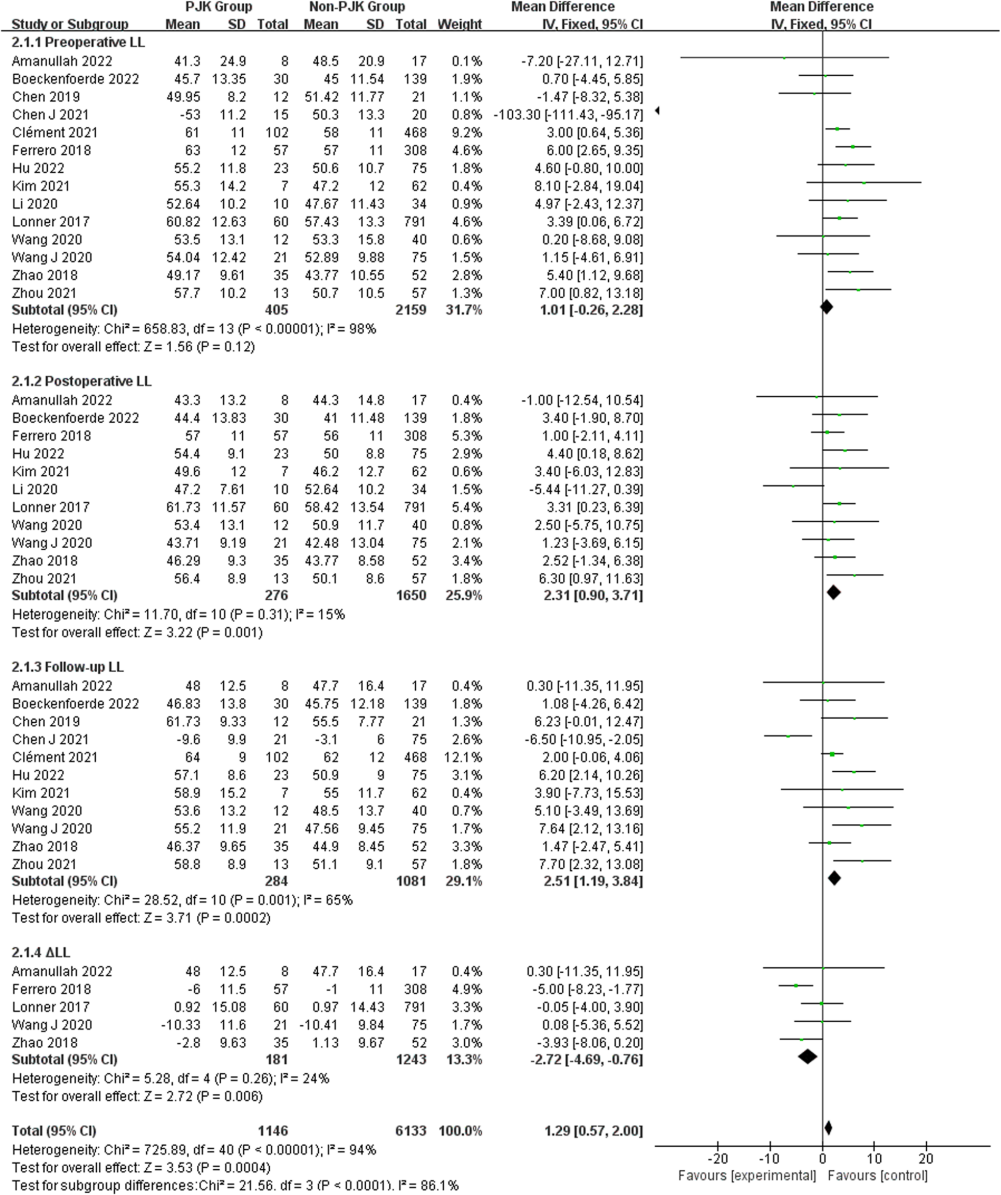
Forest plot of LL between proximal junctional kyphosis (PJK) and non-PJK groups

**Fig. 8:**
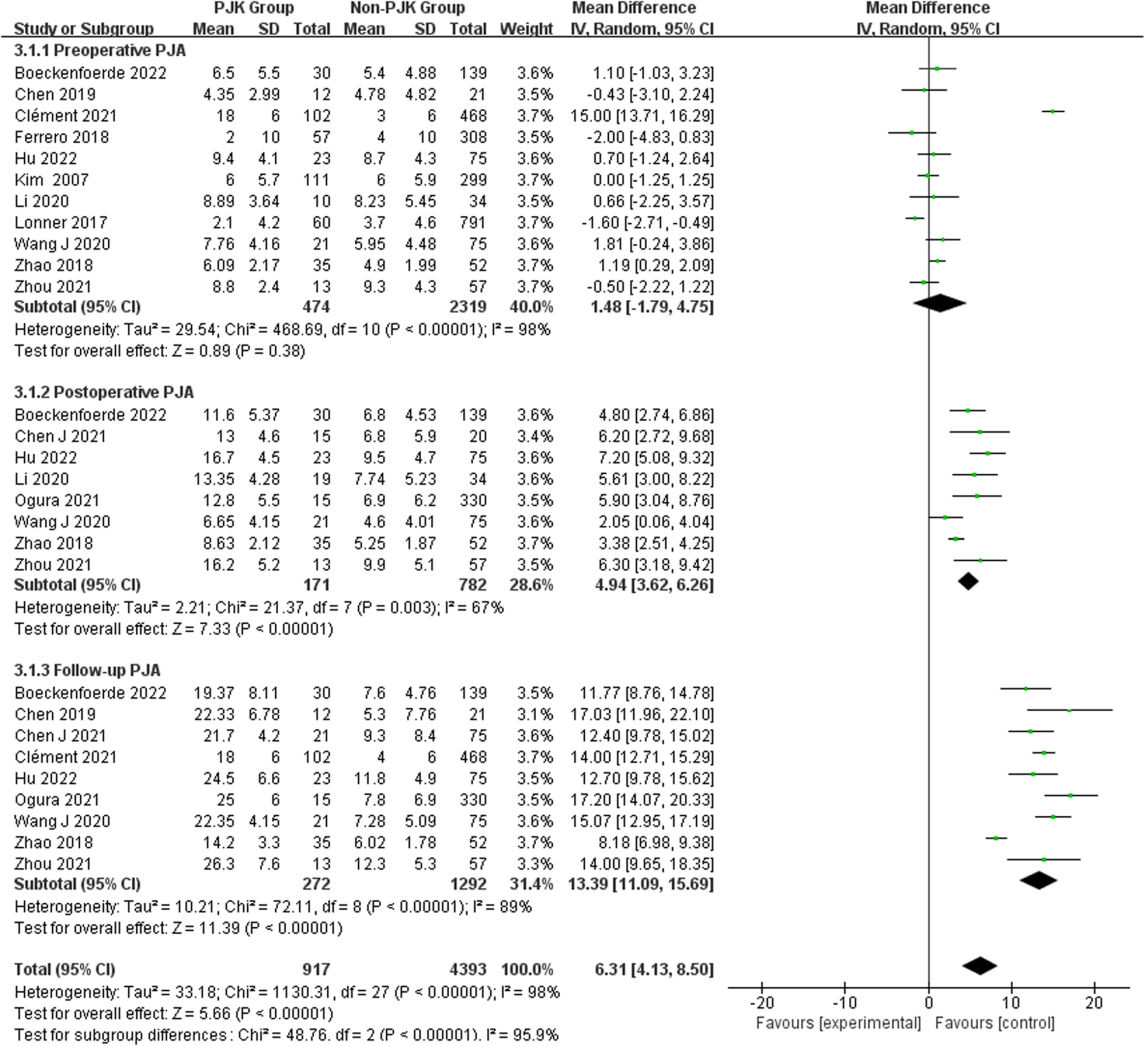
Forest plot of PJA between proximal junctional kyphosis (PJK) and non-PJK groups

**Fig. 9:**
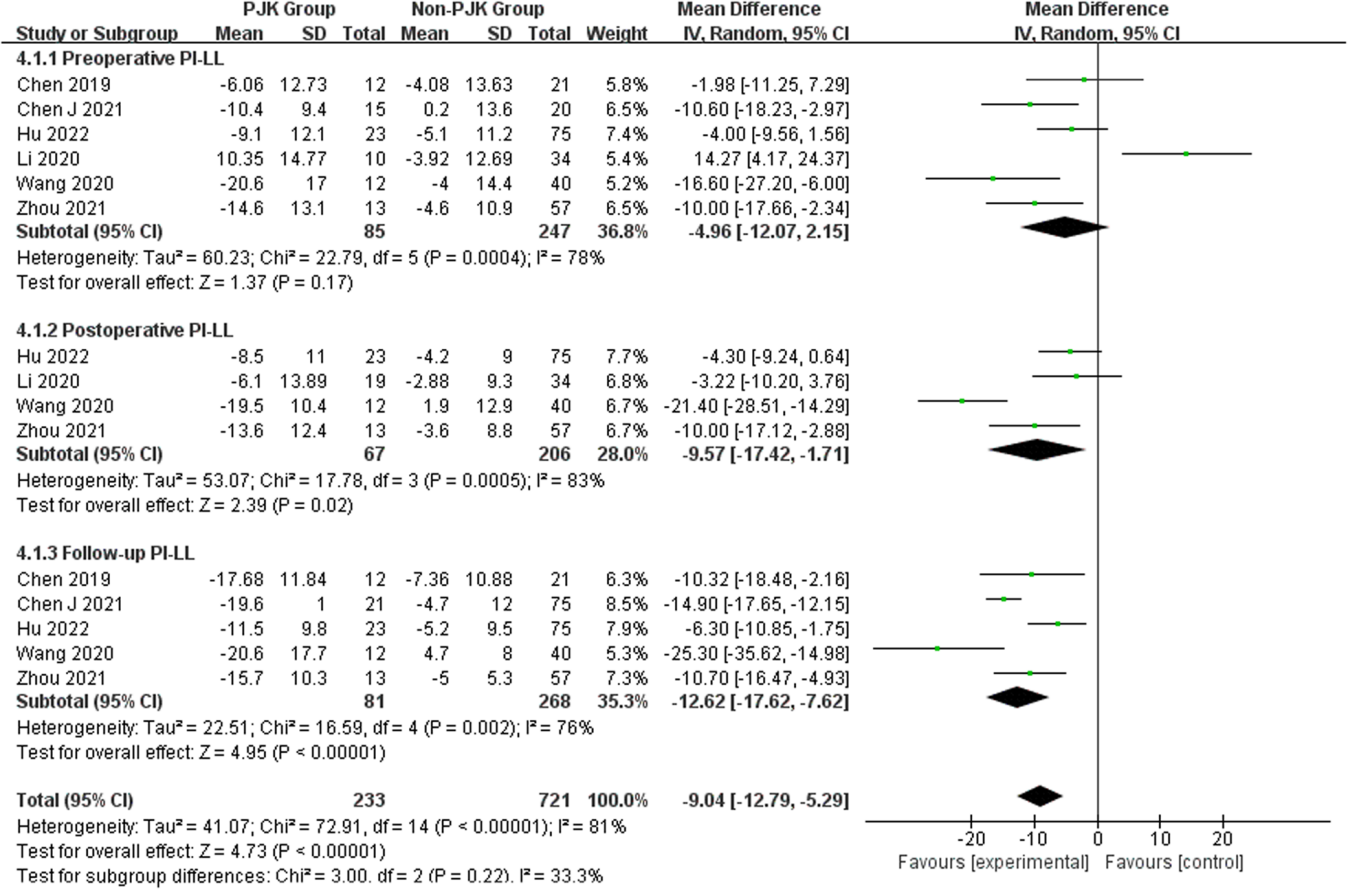
Forest plot of PI–LL between proximal junctional kyphosis (PJK) and non-PJK groups

**Fig. 10:**
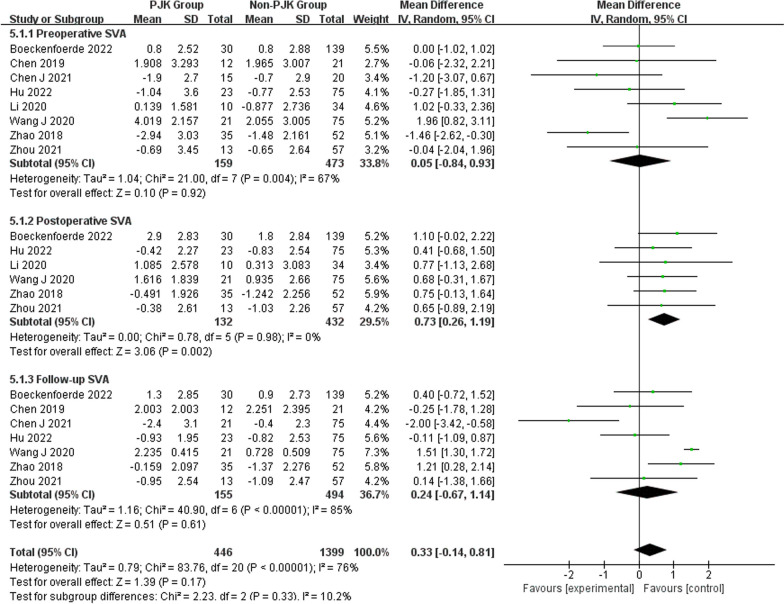
Forest plot of SVA between proximal junctional kyphosis (PJK) and non-PJK groups

**Fig. 11:**
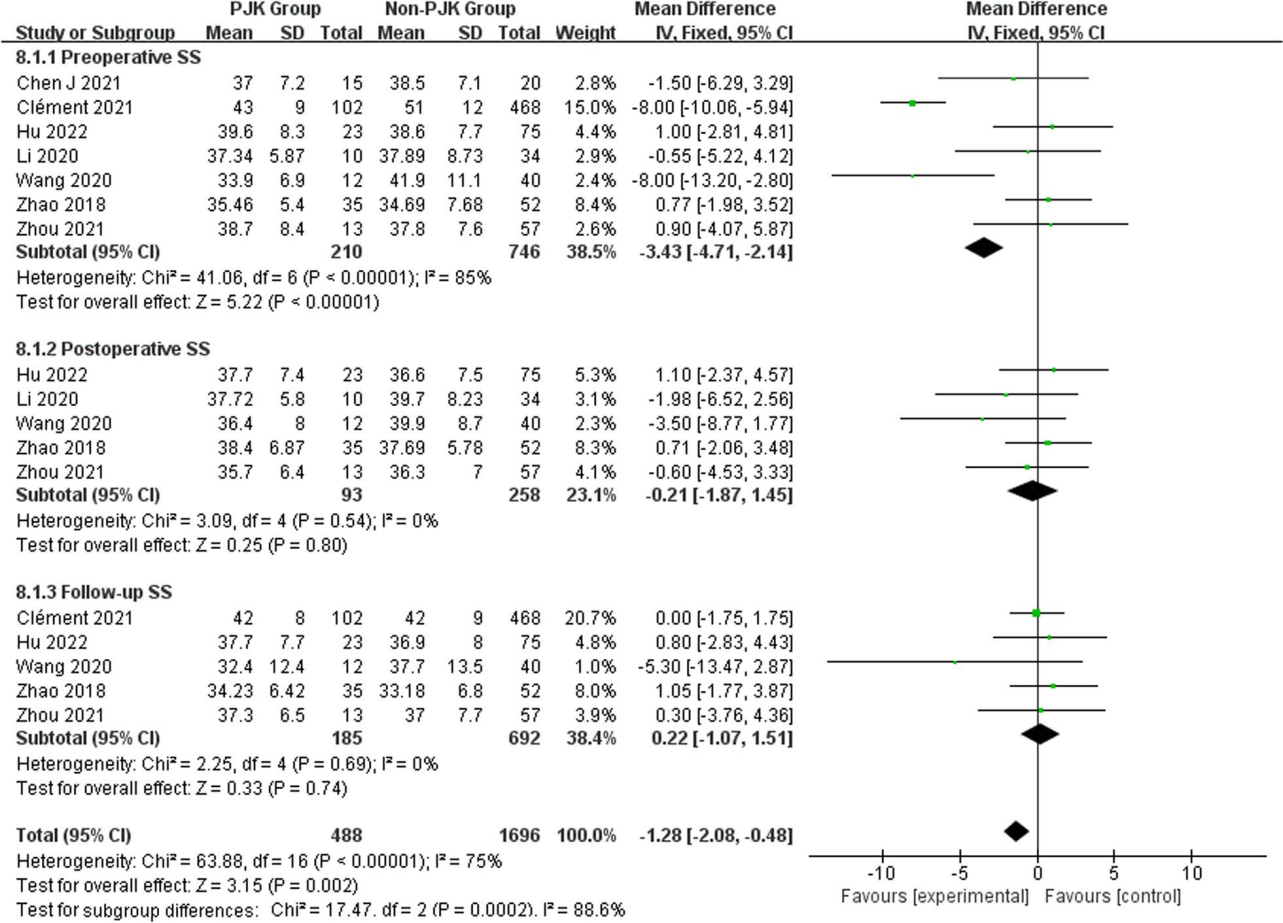
Forest plot of SS between proximal junctional kyphosis (PJK) and non-PJK groups

**Fig. 12:**
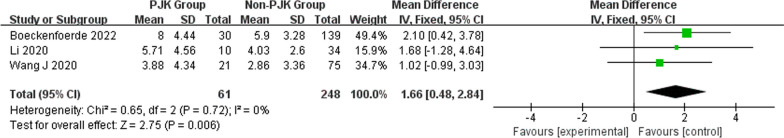
Forest plot of RCA between proximal junctional kyphosis (PJK) and non-PJK groups

Regarding radiographic parameters, meta-analysis results indicated that larger preoperative TK (WMD 6.82, 95% CI (5.48, 8.16), *P* < 0.00001) (Fig. 6), larger follow-up TK (WMD 8.96, 95% CI (5.62, 12.30), *P* < 0.00001) (Fig. 6), larger postoperative LL (WMD 2.31, 95% CI (0.91, 3.71), *P* = 0.001) (Fig. 7), larger follow-up LL (WMD 2.51, 95% CI (1.19, 3.84), *P* = 0.0002) (Fig. 7), great change in LL (WMD − 2.72, 95% CI (− 4.69, − 0.76), *P* = 0.006) (Fig. 7), larger postoperative PJA (WMD 4.94, 95% CI (3.62, 6.26), *P* < 0.00001) (Fig. 8), larger follow-up PJA (WMD 13.39, 95% CI (11.09, 15.69), *P* < 0.00001) (Fig. 8), larger postoperative PI–LL (WMD − 9.57, 95% CI (− 17.42, − 1.71), *P* = 0.02) (Fig. 9), larger follow-up PI–LL (WMD − 12.62, 95% CI (− 17.62, − 7.62), *P* < 0.00001) (Fig. 9), larger preoperative SVA (WMD 0.73, 95% CI (0.26, 1.19), *P* = 0.002) (Fig. 10), larger preoperative SS (WMD − 3.43, 95% CI (− 4.71, − 2.14), *P* < 0.00001) (Fig. 11), RCA (WMD 1.66, 95% CI (0.48, 2.84), *P* = 0.006) (Fig. 12) were identified as risk factors for PJK in patients with AIS.

**Fig. 13:**
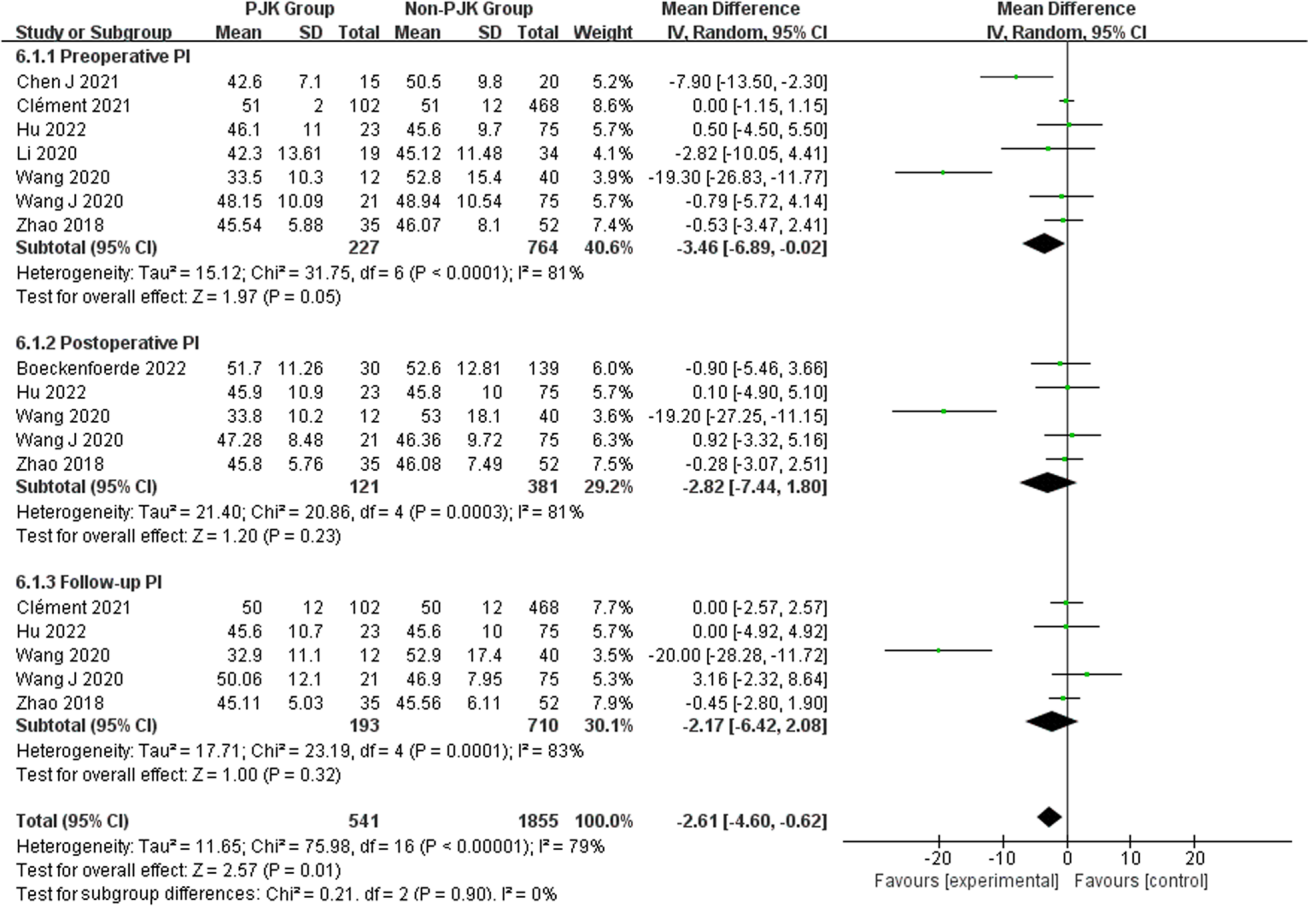
Forest plot of PI between proximal junctional kyphosis (PJK) and non-PJK groups

**Fig. 14:**
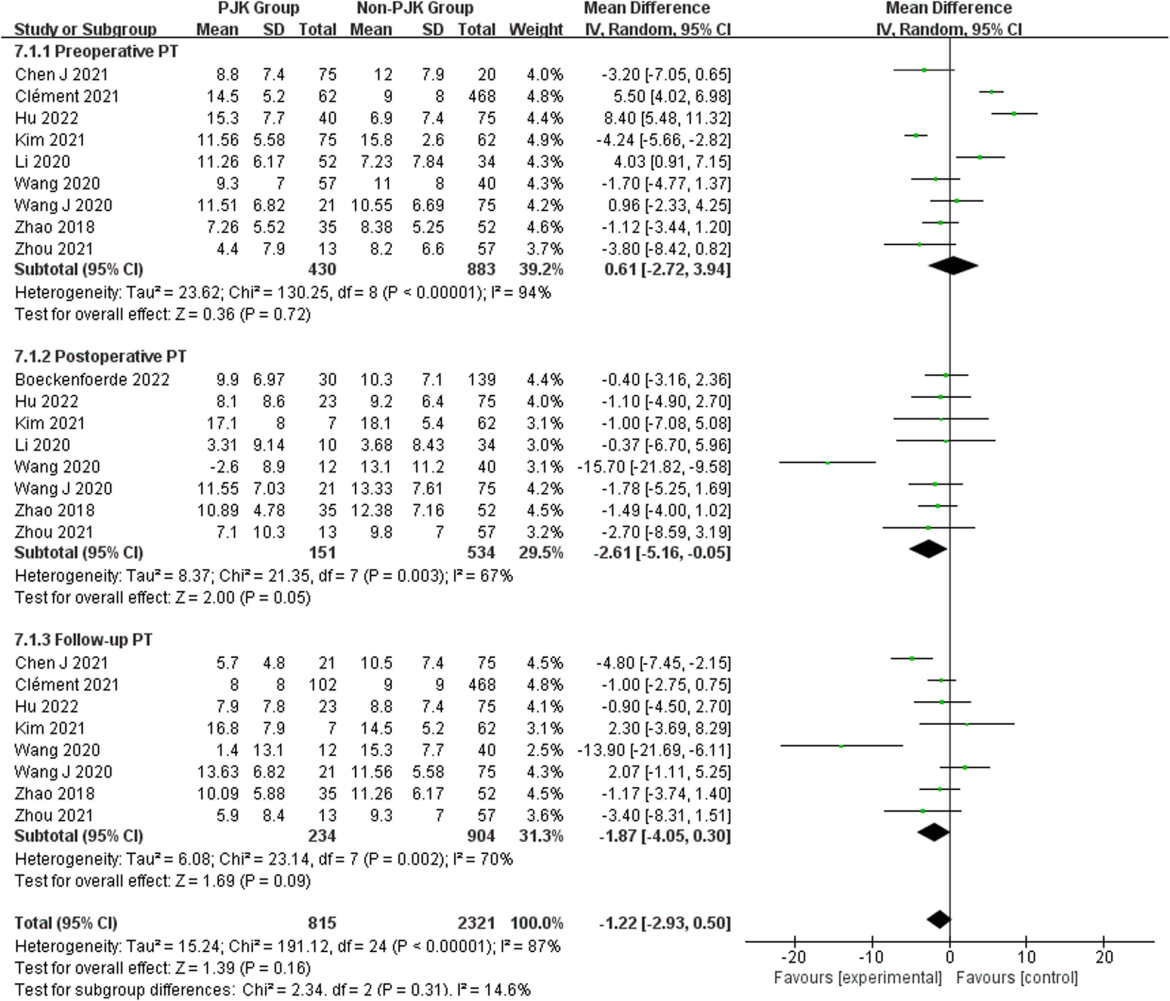
Forest plot of PT between proximal junctional kyphosis (PJK) and non-PJK groups

**Fig. 15:**
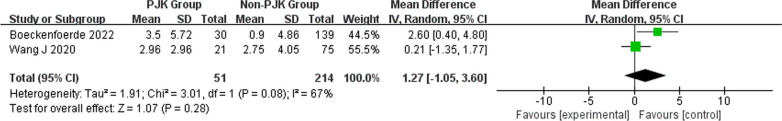
Forest plot of postoperative PJA-RCA between proximal junctional kyphosis (PJK) and non-PJK groups

**Fig. 16:**
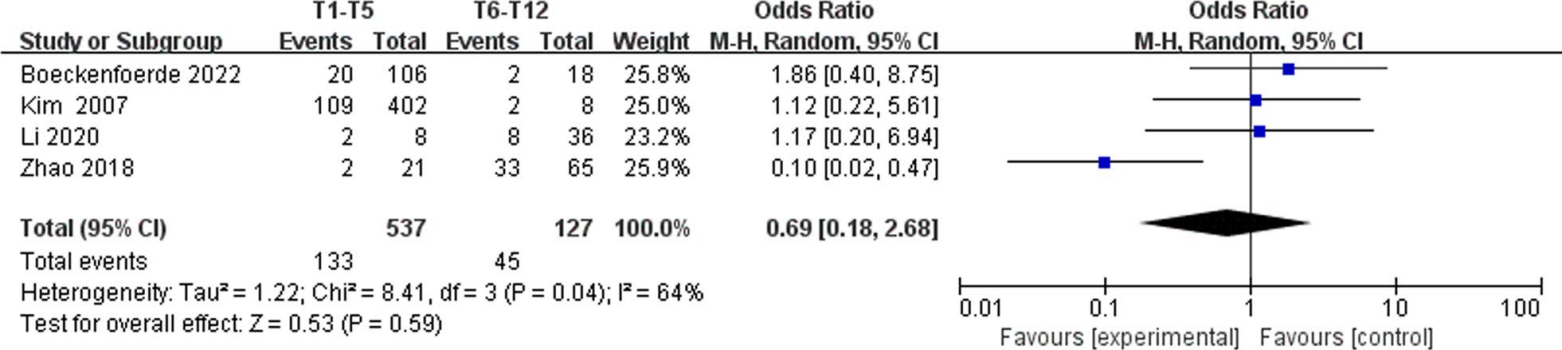
Forest plot of UIV between proximal junctional kyphosis (PJK) and non-PJK groups

However, no significant associations were discerned between postoperative TK (WMD 4.46, 95% CI (− 0.47, 9.39), *P* = 0.08) (Fig. 6), change in TK (WMD − 3.00, 95% CI (− 7.47, 1.46), *P* = 0.19) (Fig. 6), preoperative LL (WMD 1.01, 95% CI (− 0.26, 2.28), *P* = 0.12) (Fig. 7), preoperative PJA (WMD 1.48, 95% CI (− 1.79, 4.75), *P* = 0.38) (Fig. 8), preoperative SVA (WMD 0.05, 95% CI (− 0.84, 0.93), *P* = 0.92) (Fig. 10), follow-up SVA (WMD 0.24, 95% CI (− 0.67, 1.14), *P* = 0.61) (Fig. 10), preoperative PI (− 3.46 1.01, 95% CI (− 6.89, − 0.02), *P* = 0.05) (Fig. 13), postoperative PI (WMD − 2.82, 95% CI (− 7.44, 1.80), *P* = 0.23) (Fig. 13), follow-up PI (WMD − 2.17, 95% CI (− 6.42, 2.08), *P* = 0.32) (Fig. 13), preoperative PT (WMD 0.61, 95% CI (− 2.72, 3.94), *P* = 0.72) (Fig. 14), postoperative PT (WMD − 2.61, 95% CI (− 5.16, − 0.05), *P* = 0.05) (Fig. 14), follow-up PT (WMD − 1.87, 95% CI (− 4.05, 0.30), *P* = 0.09) (Fig. 14), preoperative PI–LL (WMD − 4.96, 95% CI (− 12.07, 2.15), *P* = 0.17) (Fig. 9), postoperative SS (WMD − 0.21, 95% CI (− 1.87, 1.45), *P* = 0..80) (Fig. 11), follow-up SS (WMD 0.22, 95% CI (− 1.07, 1.51), *P* = 0.74) (Fig. 11), postoperative PJA-RCA (WMD 1.27, 95% CI (− 1.05, 3.60), *P* = 0.28) (Fig. 15), UIV (WMD 0.69, 95% CI (0.18, 2.68), *P* = 0.59) (Fig. 16) and occurrence of PJK.

### Subgroup analysis

**Fig. 17:**
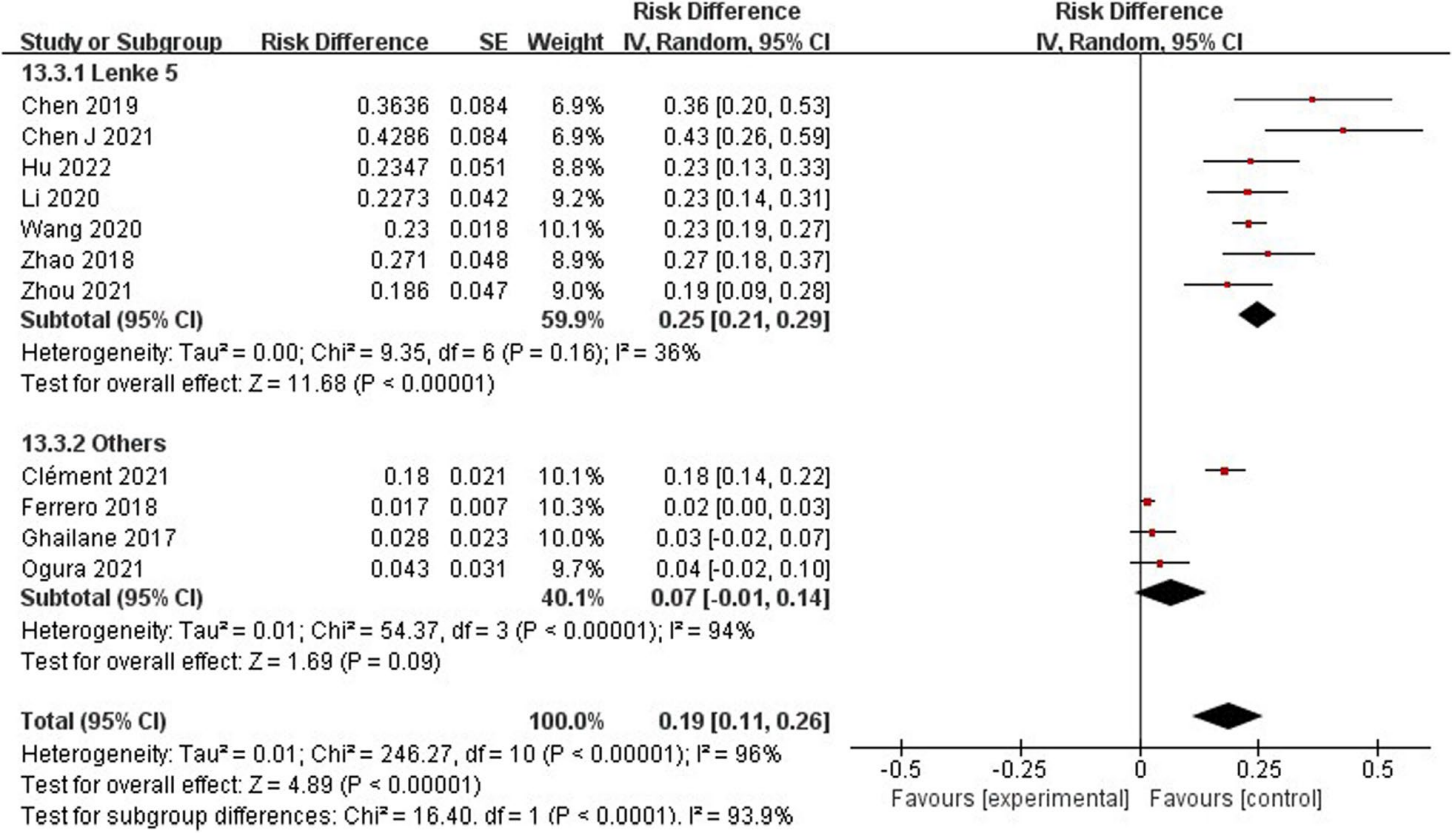
Subgroup analysis of pooled incidence of proximal junctional kyphosis

**Fig. 18:**
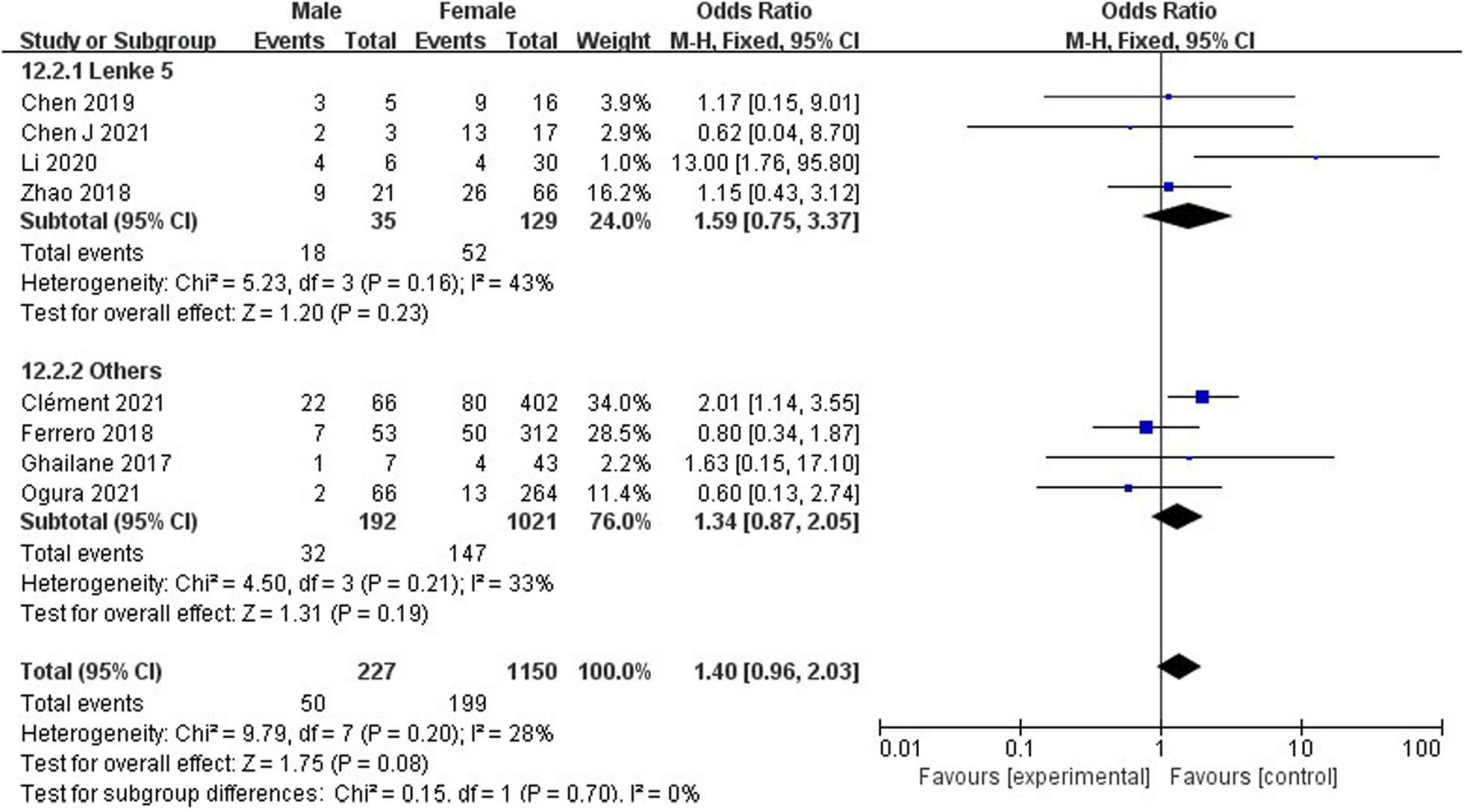
Subgroup analysis of forest plot of proximal junctional kyphosis between the male and female groups

According to the subgroup analysis of AIS classification, it was found that the probability of occurrence of PJK in Lenke 5 type (25%, 95% CI 21–29%) (Fig. 17) was significantly higher than that in other types. Sex in the subgroup (Fig. 18) was not a risk factor for PJK after Lenke 5 AIS. Age (WMD − 0.37, 95% CI (− 0.81, 0.07), *P* = 0.10) (Additional file 1: Figure S1) was not a risk factor for postoperative PJK.

Regarding radiographic parameters, meta-analysis results indicated that larger preoperative TK (WMD 7.85, 95% CI (5.69, 10.00), *P* < 0.00001) (Additional file 1: Figure S2), larger postoperative TK (WMD 9.66, 95% CI (1.06, 18.26), *P* = 0.03) (Additional file 1: Figure S2), larger follow-up TK (WMD 11.92, 95% CI (6.99, 16.86), *P* < 0.00001) (Additional file 1: Figure S2), larger preoperative PJA (WMD 0.72, 95% CI (0.03, 1.41), *P* = 0.04) (Additional file 1: Figure S4), larger postoperative PJA (WMD 5.54, 95% CI (3.57, 7.52), *P* < 0.00001) (Additional file 1: Figure S4), larger follow-up PJA (WMD 12.42, 95% CI 9.24, 15.60), *P* < 0.00001) (Additional file 1: Figure S4), larger follow-up SVA (WMD 0.07, 95% CI (− 0.46, 0.60), *P* = 0.04) (Additional file 1: Figure S5), larger preoperative PT (WMD − 3.04, 95% CI (− 5.27, − 0.81), *P* = 0.008) (Additional file 1: Figure S7), larger follow-up PT (WMD − 3.69, 95% CI (− 6.66, − 0.72), *P* = 0.02) (Additional file 1: Figure S7) were identified as risk factors for PJK in patients with Lenke 5 AIS.

However, no significant associations were discerned between larger preoperative LL (WMD –11.72, 95% CI (− 36.09, 12.64), *P* = 0.35) (Additional file 1: Figure S3), larger postoperative LL (WMD 2.25, 95% CI (− 1.40, 5.90), *P* = 0.23) (Additional file 1: Figure S3), larger follow-up LL (WMD 3.14, 95% CI (− 1.46, 77.74), *P* = 0.18) (Additional file 1: Figure S3), preoperative SVA (WMD − 0.41, 95% CI (− 1.05, 0.23), *P* = 0.21) (Additional file 1: Figure S5), follow-up SVA (WMD 0.07, 95% CI (− 0.46, 0.60), *P* = 0.79) (Additional file 1: Figure S5), preoperative PI (WMD − 5.62, 95% CI (− 11.80, 0.56), *P* = 0.07) (Additional file 1: Figure S6), postoperative PI (WMD − 5.66, 95% CI (− 14.60, 3.28), *P* = 0.21) (Additional file 1: Figure S6), follow-up PI (WMD − 5.89, 95% CI (− 14.69, 2.92), *P* = 0.19) (Additional file 1: Figure S6), postoperative PT (WMD − 3.95, 95% CI (− 8.43, 0.53), *P* = 0.08) (Additional file 1: Figure S7), preoperative SS (WMD − 0.49, 95% CI (− 2.14, 1.16), *P* = 0.56) (Additional file 1: Figure S8), postoperative SS (WMD − 0.21, 95% CI (− 1.87, 1.45), *P* = 0.80) (Additional file 1: Figure S8), follow-up SS (WMD 0.47, 95% CI (− 1.42, 2.37), *P* = 0.62) (Additional file 1: Figure S8) and occurrence of PJK.

### Sensitivity analyses and publication bias

**Fig. 19:**
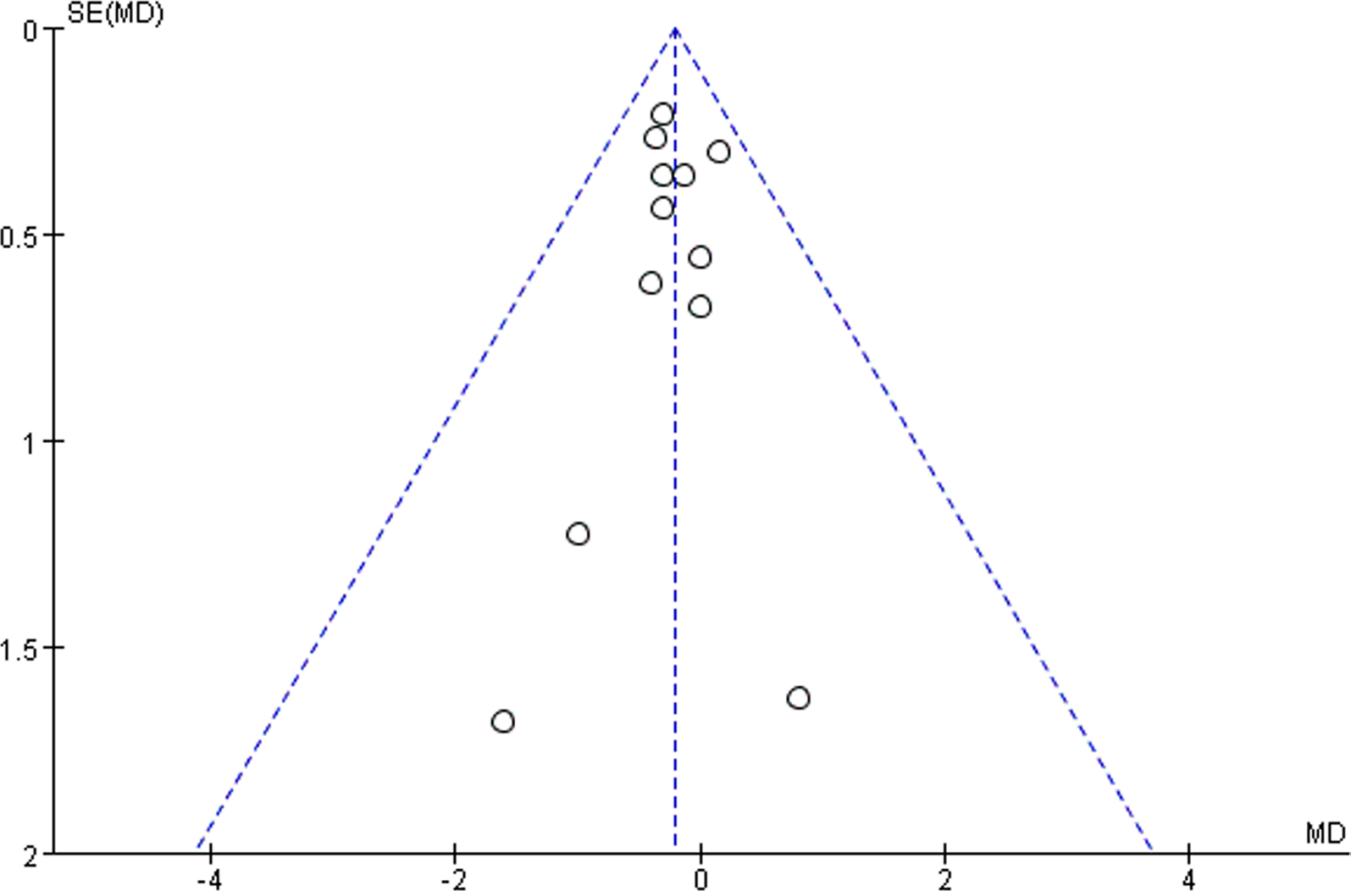
Risk of publication bias in the included literature

Sensitivity analysis was carried out by individually calculating and subtracting each study from the meta-analysis in order to ascertain the impact of each one. Publication bias was screened using funnel plots. A *P* < 0.05 was considered statistically significant. An example is indicated by sensitivity analysis showing the funnel plot of age reported in this meta-analysis for PJK and non-PJK groups (Fig. 19). Any study could be excluded after the heterogeneity test without significantly changing the overall statistical significance, showing that the findings of this meta-analysis were stable. Additionally, the funnel plot's shape was symmetrical, indicating that our study did not contain publication bias.

## Discussion

The incidence of PJK in patients with AIS was 19%. Before the typing comparison was performed, this meta-analysis found that sex, larger preoperative TK, larger follow-up TK, larger postoperative LL, larger follow-up LL, great LL change, larger postoperative PJA, larger follow-up PJA, larger postoperative PI–LL, larger follow-up PI–LL, larger preoperative SVA, larger preoperative SS and RCA were identified as risk factors for PJK in AIS after correction surgery.

A frequent side effect of spinal deformity surgery is PJK. Numerous factors, including demographic, surgical, and radiological parameters, contribute to the development of PJK. Patients with AIS undergo orthopedic surgery to reconstruct coronal and sagittal alignment to maintain spinal stability [32]. Acute proximal junctional kyphosis can be caused by a fracture of the UIV during the chronic course or by deformation of the interspinous ligament and facet joint components at the level of the UIV [33]. The occurrence of PJK has been described as a compensatory mechanism [34] and may result from the postoperative imbalance caused by increased lumbar lordosis (LL), insufficient TK, or a mismatch in thoracolumbar alignment [18, 35]. Regardless of the imaging criteria, PJK can become pathological and lead to proximal junctional failure (PJF) [35], causing pain, neurological dysfunction, and deformity progression, and even requiring secondary surgery.

Increasing age can be counted as an important risk factor [36]. The severity of PJK increased with the increase of corrected age. This study did not identify age as a risk factor for PJK. This study mainly included adolescents, so the effect of age on PJK has not been reflected. Further subgroup analysis did not find that age was a risk factor for PJK, either. Initially, Kim found that [37] the male gender was associated with PJK. However, this study verified that the incidence of PJK in women was higher than that in men, which was different from a meta-analysis in 2019 [38] which had not yet found a role for gender in PJK. We hypothesized that women are the risk factors for PJK in AIS, which may be related to the natural anatomy of women, with larger thoracolumbar Angle and greater probability of AIS occurrence [39]. However, gender was not found to be a risk factor for PJK after typing analysis. Due to data limitations, not all studies performed gender subgroup analysis, so this conclusion is disputed. Patient-specific factors, such as obesity, are important considerations before any spinal surgery [7]. These findings do not support that BMI was a risk factor for PJK. The inclusion criteria were likely put in place to allow for group comparison, and further studies are needed to observe whether the incidence of PJK can be improved by controlling body weight. The above results are similar to the conclusions of Peng et al. [40], who found no statistically significant difference in age at surgery and BMI. Zhao et al. [41] also came to a similar conclusion. The current study's findings are generally consistent with earlier findings in terms of these demographic factors.

The relationship between TK, LL, and the incidence of PJK was first examined. It was found that large preoperative TK, large postoperative follow-up TK, postoperative LL, large postoperative follow-up LL and the change of LL were the risk factors of PJK. It is hypothesized that an excessively large TK Angle and an excessive amount of LL correction will increase the prevalence of PJK. The results of Lonner et al. [25] found that the preoperative TK of the PJK group was significantly higher than that of the non-PJK group. Further, logistic regression analysis confirmed that for every 10-degree increase in TK, the risk of PJK increased by 6%. Both Kim and Lafage [42, 43] found a higher incidence of PJK with more corrected LL, and they considered surgical overcorrection as a risk factor. The patient can regain balance by reducing proximal thoracic kyphosis and/or increasing distal lumbar lordosis following surgical repair of AIS, which causes the rebalancing phenomena known as PJK [17] thus increasing the burden of LL. Strong surgical TK correction does not encourage PJK, but it is beneficial in playing a small compensatory role in mild LL correction. Moreover, Kim et al. [42] found that excessive lordosis and large sagittal balance correction resulted in PJK, which required revision surgery.

The results show that pelvic parameters, such as PI, PT, and SS, are significant factors that must be considered while researching spinal morphology and balance. Pelvic incidence (PI) is a parameter that truly reflects the pelvic anatomy. PT, is an indicator of the compensation degree of spinal deformity, and SS is recognized as an important determinant of lumbar lordosis angle (LL). All three factors together affect the sagittal spinal morphology of AIS. According to studies, aberrant PI may increase the chance of sagittal malalignment following scoliosis fusion surgery, lowering the quality of life and aggravating symptoms [44]. Annis et al. [45] identified elevated PI and pelvic retroversion as factors that increase the risk of PJK. However, the conclusions of this study have not confirmed the separate association between PI and PT, and PJK. Zhao et al. [41] also found no significant difference in pelvic parameters between the PJK and non-PJK groups; however, they also reported that the association between pelvic parameters and PJK could not be ignored during long-term follow-up. Emmanuelle et al. [18] found that patients with high PI compensated for sagittal imbalance by pelvic reverse tilt; therefore, they were at higher risk of PJK. However, no subgroup analysis of PI was performed in this study. According to several studies, adults who are pathologically involved and asymptomatic show a substantial correlation between PI and LL [46]. Wang et al. demonstrated [47] that restoring the ideal postoperative PI–LL relationship can reduce the PJK rate. Moreover, this study also found that small postoperative PI–LL and follow-up PI–LL were risk factors for PJK. It has been suggested that maintaining a specific degree of curvature between the lumbar spine and pelvis following surgery can significantly lower the incidence of PJK. Additionally, PJK risk is also increased by reduced preoperative SS. These findings are partially consistent with the conclusions of Annis et al. [45].

Sagittal anteversion is exacerbated by spinal deformity in AIS, which is balanced by a variety of pelvic factors. The sagittal vertical axis makes it simple to gauge this sagittal imbalance (SVA). A positive sagittal alignment indicates a decompensated mechanism, which gradually advances to low back pain and impaired lung function. This study identified large postoperative SVA as a risk factor for PJK, which is not consistent with the conclusions drawn by Wang [29] and Burton et al. [48]. It is considered that the reason may be the heterogeneity of the population or the severity of the deformity, or the influence of other pelvic parameters. SVA does not, however, enhance the likelihood of PJK after follow-up, most likely due to the compensating function of the spine, which in turn complements the sagittal imbalance.

In a study of 87 cases, Zhao et al. [30] observed that increased postoperative PJA was a major risk factor for PJK in Lenke type 5 AIS patients. In this study, there was a correlation between large postoperative PJA and the incidence of follow-up PJA and PJK. Preoperative PJA over 5° has been reported as a risk factor for PJK [49]. Further evidence from biomechanical studies by Cammarata et al. [50], demonstrated that an increase in RCA from 10° to 20°, 30°, and 40° increased PJA by 6%, 13%, and 19%, suggesting that inappropriate bending of an overbent sagittal rod produces PJK. Wang et al. [29] found that the occurrence of PJK should be highly considered in patients with preoperative PJA-RCA greater than 5°. Boeckenfoerde et al. [14] found that high preoperative RCA and increased postoperative PJA-RCA differences were risk factors for PJK. This study also found that high preoperative RCA was a risk factor for PJK, but postoperative PJA-RCA was not associated with the occurrence of PJK. However, due to the small number of included studies, the change in the difference warrants further study. Currently, the majority of studies concentrate on the value of sagittal bar profiles in PJK prevention. To reestablish the proper sagittal equilibrium of the spine, sustained attention should be given to the angle's change in the future.

The study by Li et al. [24] showed that UIV not in the lower thoracic spine was a risk factor of PJK. In this study, the choice of the upper and lower thoracic vertebrae of the UIV did not reflect the difference, which was inconsistent with the results of previous studies. Data collection was limited in the included studies, which may be due to the differences in distinguishing segments, so the conclusions of the studies are controversial.

Correcting the total spinal alignment and balance following surgery can minimize PJK with the use of TK, LL, and PI [51]. Reduced thoracic kyphosis induces reduced cervical and lumbar lordosis to achieve longitudinal stability [52]. In accordance with various PI values, the correction range of the LL should be precisely measured and planned prior to surgery to prevent overcorrection. Additionally, the changes in the PJA, SVA, SS, and RCA should be monitored concurrently to minimize the occurrence of proximal junctional kyphosis (PJK) and restore normal spinal cord balance to maximize functional outcomes and relieve pain [53].

In our research, we discovered that Lenke 5 AIS sufferers faced a greater risk of PJK formation post-orthopedic surgery compared to others. Strikingly, LL did not pose an increased risk for PJK. This result contrasts with the general findings. This finding suggests that different patient types require distinct considerations. For Lenke 5 AIS patients, the spotlight fell on TK indicators. Considering the limited number of indicators for other types of AIS in the existing literature, a meta-analysis was challenging to pinpoint the corresponding indicators. Thus, understanding the risk factors leading to PJK postoperatively becomes more specific when considering treatment based on Lenke's typology.

## Limitations

All included studies were retrospective. In this paper, the classification of different types of AIS patients was not studied because the classification was not very clear in the included studies. The postoperative SRS-22 score was only briefly discussed in the literature, and this study did not perform subgroup analysis of age, BMI and PI, did not collect various types of spinal deformity, did not include surgical methods like lower fixation cone (LIV), or whether to perform derotation, osteotomy or thoracoplasty. Recently, the attention to screw hook and screw fixation has decreased, and most studies have not mentioned this aspect, so this paper does not conduct a comprehensive analysis.

## Conclusion

In this study, we found the incidence of PJK in patients with AIS was 19% after correction surgery, while Lenke 5 is seen in 25%. Future studies could delve into finding the imaging characteristics specific to AIS for enhancing TK correction and preventing overcorrection of LL. Special focus on Lenke type could be beneficial as it can steer pre-operative planning, surgical execution, and potentially helping prevent PJK.

### Supplementary Information

**Additional file 1.** The subgroup analysis of AIS classification.

## Data Availability

All data generated or analysed during this study are included in this published article.

## Abbreviations

PJK: Proximal junctional kyphosis
PJF: Proximal junctional failure
UIV: Upper instrumented vertebra
TK: Thoracic kyphosis
LL: Lumbar lordosis
PJA: Proximal junctional angle
SVA: The sagittal vertical axis
PI: Pelvic incidence
PT: Pelvic tilt
PI–LL: Pelvic incidence–lumbar lordosis
SS: Sacral slope
RCA: Rod contour angle
MPR: Multiple pregnancy rate
95%CI: 95%confidence interval
RR: Risk ratio
WMDs: Weighted mean differences
PROSPERO: International prospective register of systematic reviews
PRISMA: Preferred Reporting Items for Reviews and Meta-Analyses
