# Supplementary material for: Incidence and risk factors of proximal junctional kyphosis in adolescent idiopathic scoliosis after correction surgery: a meta-analysis and systematic review
Source: J Orthop Surg Res. 2024 Apr 2;19:217. doi: 10.1186/s13018-024-04638-7 (PMC10988869; doi:10.1186/s13018-024-04638-7)

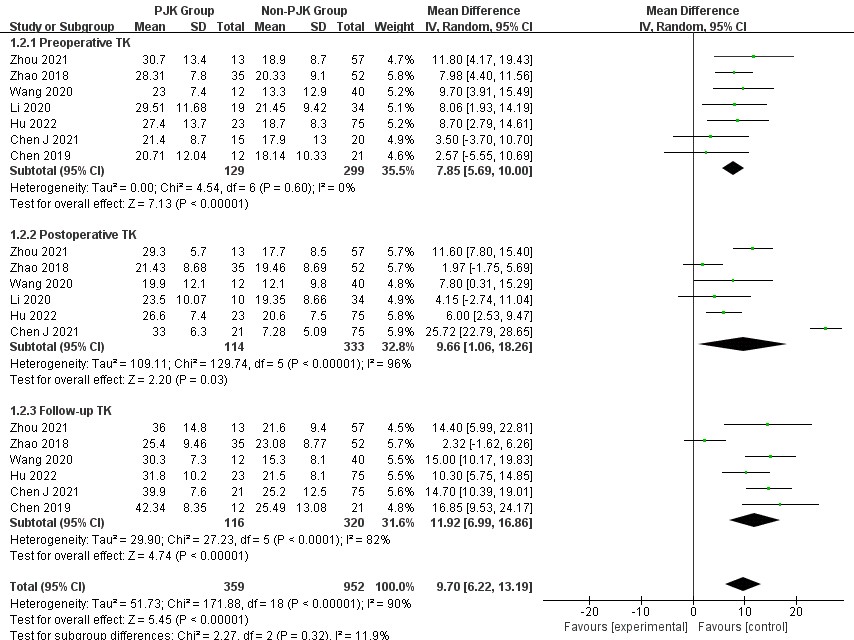

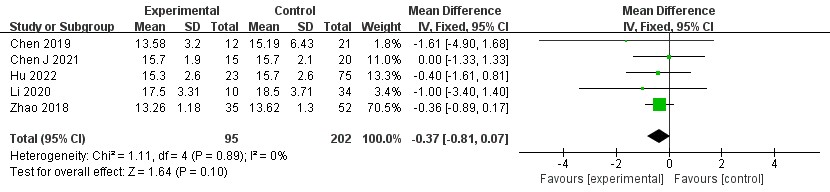
 Supplementary figure 1 Subgroup analysis of forest plot of age between the proximal junctional kyphosis (PJK) group and the non-PJK.

Supplementary figure 2 Subgroup analysis of forest plot of TK between proximal junctional kyphosis (PJK) and non-PJK groups.


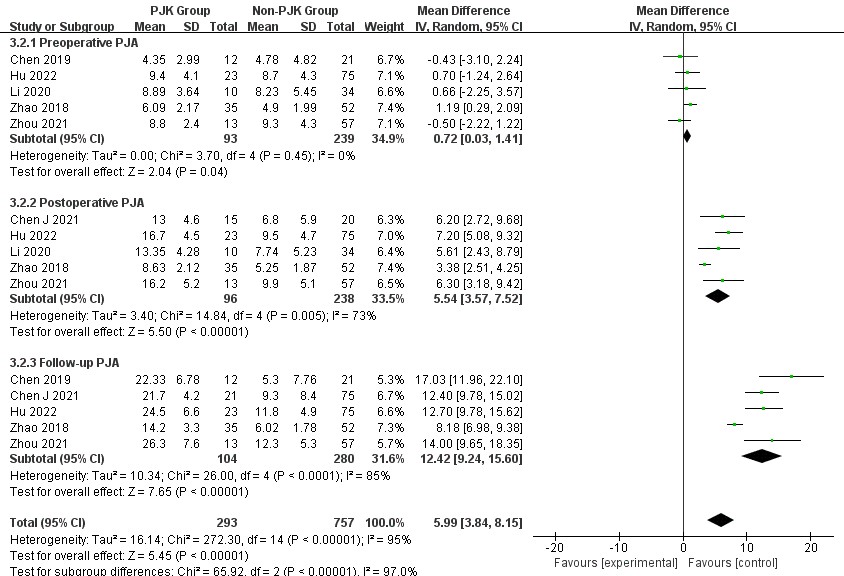

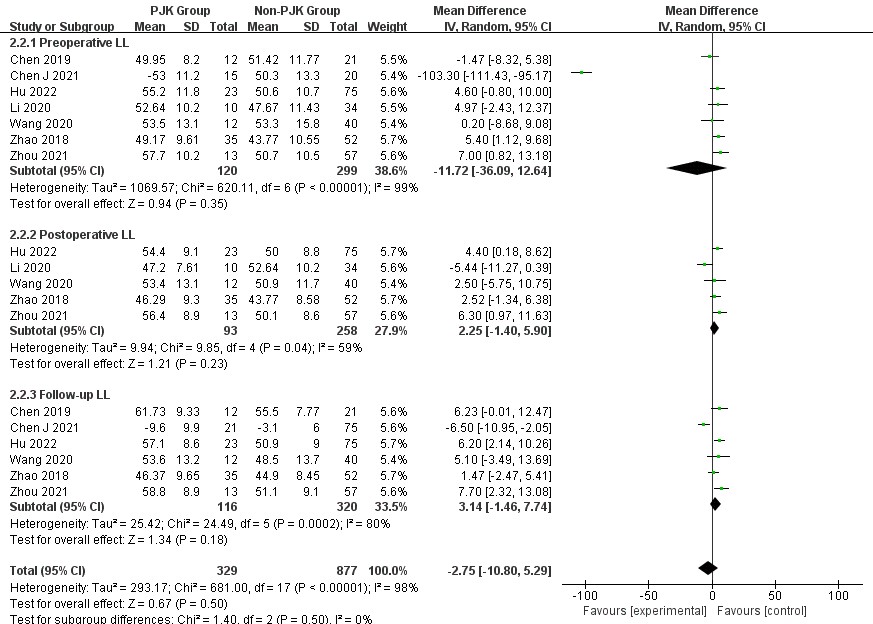
Supplementary figure 3 Subgroup analysis of forest plot of LL between proximal junctional kyphosis (PJK) and non-PJK groups.

Supplementary figure 4 Subgroup analysis of forest plot of PJA between proximal junctional kyphosis (PJK) and non-PJK groups.


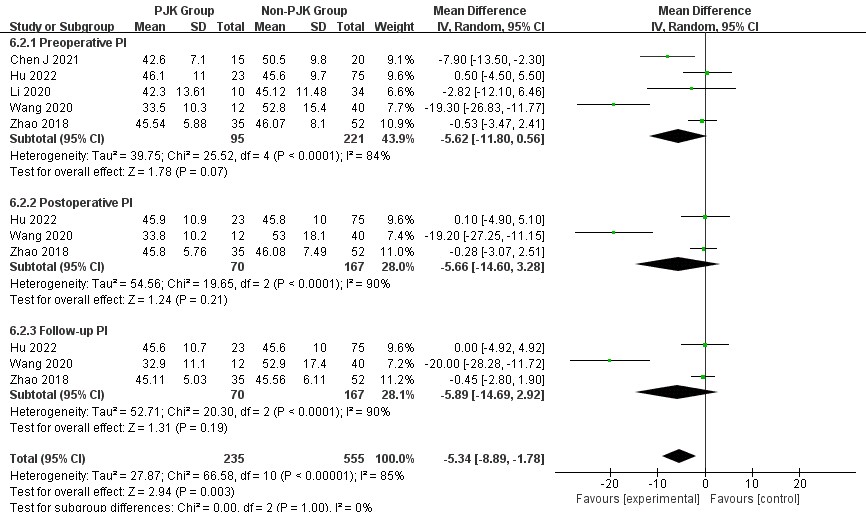

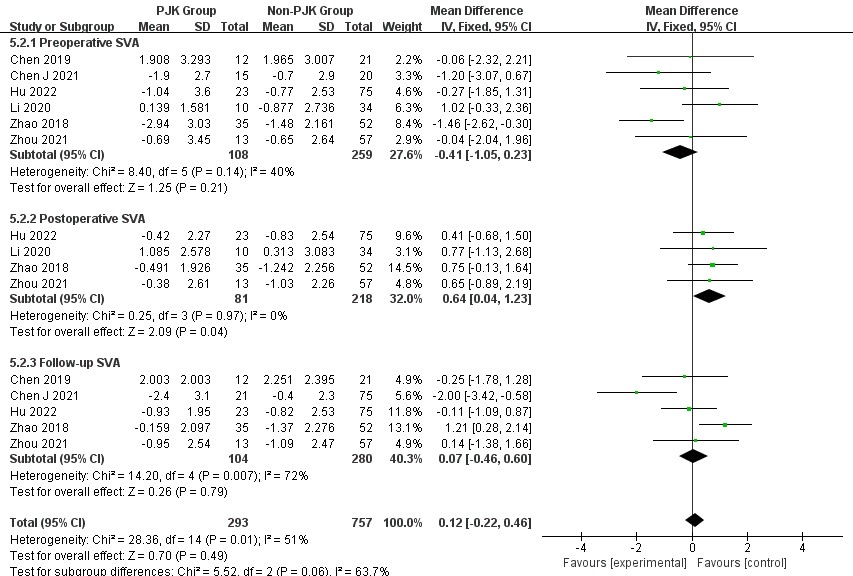
Supplementary figure 5 Subgroup analysis of forest plot of SVA between proximal junctional kyphosis (PJK) and non-PJK groups.

Supplementary figure 6 Subgroup analysis of forest plot of PI between proximal junctional kyphosis (PJK) and non-PJK groups.


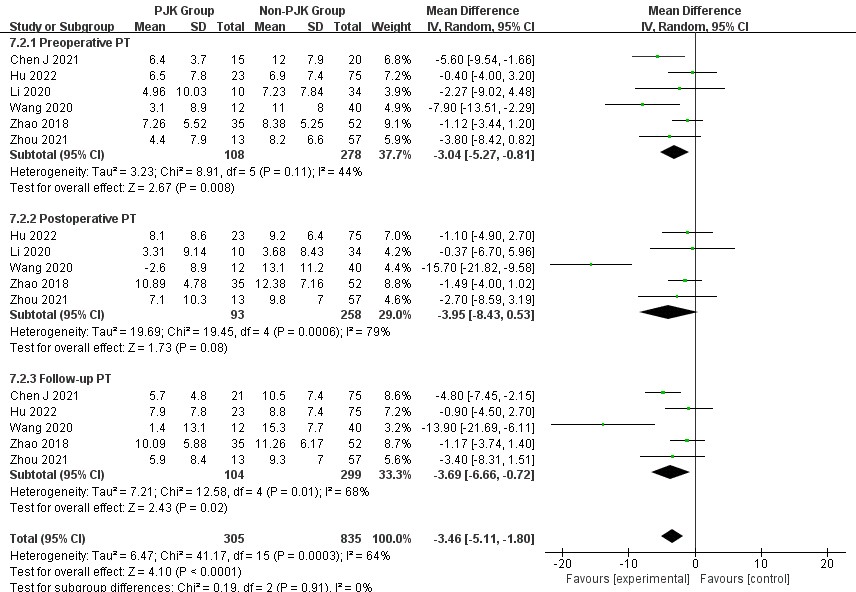
Supplementary figure 7 Subgroup analysis of forest plot of PT between proximal junctional kyphosis (PJK) and non-PJK groups.

Supplementary figure 8 Subgroup analysis of forest plot of SS between proximal junctional kyphosis (PJK) and non-PJK groups.
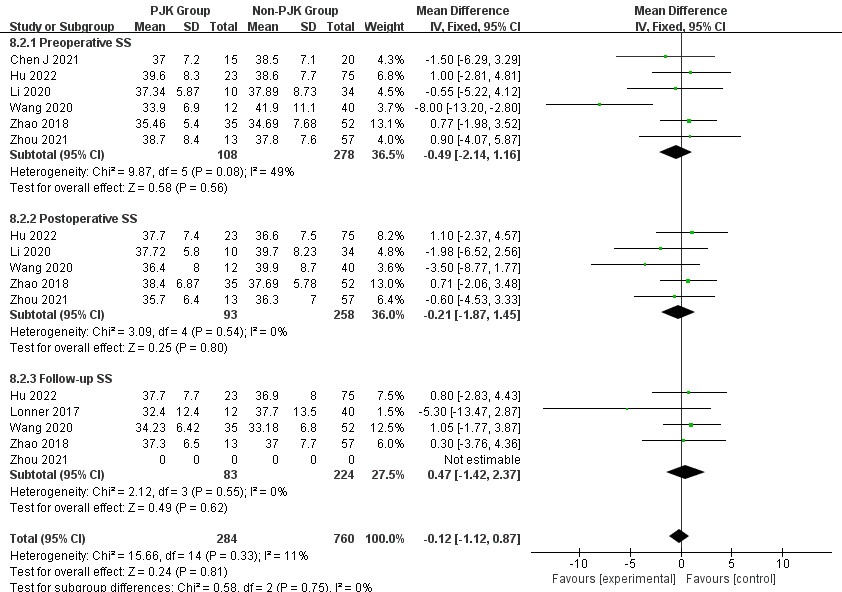

Supplement: Supplementary file 1 — Additional file 1. The subgroup analysis of AIS classification. [file 13018_2024_4638_MOESM1_ESM.docx]
